# Sexual dimorphism of Malaysian Mahseer, *Tor tambroides* broodstock reared in captivity: Morphometric measurements dataset

**DOI:** 10.1016/j.dib.2020.106557

**Published:** 2020-11-21

**Authors:** Muhammad Yazed Abduh, Nor Hakim Norazmi-Lokman, Mohammad Syahnon, Gusti Afiz Gusti Roslan, Nurul Hayati Ismail, Ambok Bolong Abol-Munafi

**Affiliations:** a Faculty of Fisheries and Food Sciences, Universiti Malaysia Terengganu, 21030 Kuala Nerus, Terengganu, Malaysia; b Institute of Tropical Aquaculture and Fisheries, Universiti Malaysia Terengganu, 21030 Kuala Nerus, Terengganu, Malaysia; c Fisheries and Aquaculture Centre, Institute for Marine and Antarctic Studies, 7053 Taroona, Tasmania, Australia

**Keywords:** Aquaculture, Conservation, ImageJ, Truss network, Tropical fish

## Abstract

Despite having high economical value and declining wild population, the aquaculture of the Malaysian mahseer (*Tor tambroides*) is still way behind. Crucial information such as on its sexual dimorphism is scarce thus making its broodstock management in captivity challenging. The first step in studying fish sexual dimorphism is by observing and identifying the morphological differences between each sex. Thus, this article collected and described morphometric measurements data of broodstock reared in captive conditions. The broodstock were reared and raised in captivity for five years since they were bought from a local commercial supplier while they were five-inch fingerlings. Seven conventional and 21 Truss network morphometric measurements were taken from 27 male (TL: 53.88 ± 2.86 cm; W: 1.82 ± 0.23 kg) and 27 female (TL: 53.97 ± 3.62 cm; W: 1.86 ± 0.35 kg) *T. tambroides* broodstocks using digital image analysis. Photograph of the fish broodstocks were captured and the morphometric measurements were conducted using imageJ freeware. Statistical analysis was later conducted on the morphometric data to identify the morphological differences between the broodstocks. This dataset will not only aid the sex identification of this species but also in stock population studies thus accelerating the development of *T. tambroides* aquaculture while improving its conservation efforts.

## Specifications Table

**Table:** 

Subject	Aquatic science
Specific subject area	Aquaculture
Type of data	Table Figure
How data were acquired	Data were acquired by capturing and analyzing digital image male and female broodstocks reared in captivity for 5 years. Morphometric measurements were quantified using ImageJ freeware followed by statistical analysis IBM SPSS statistics software version 25 (IBM, US).
Data format	Raw Analysed
Parameters for data collection	Standard morphometric and Truss network system measurements.
Description of data collection	A total of 28 morphometric characteristic measurements were taken from 27 male and female *Tor tambroides* broodstocks respectively reared in captivity for five years.
Data source location	Institution: Faculty of Fisheries and Food Sciences City/Town/Region: Kuala Terengganu, Terengganu Country: Malaysia Latitude and longitude (and GPS coordinates, if possible) for collected samples/data: Mahseer Hatchery, Faculty of Fisheries and Food Sciences, Universiti Malaysia Terengganu: 5°24′34.2"N 103°05′22.6"E
Data accessibility	1 With the article 2 Repository name: Mendeley Data Data identification number: DOI: 10.17632/c3pvc272df.2 Direct URL to data: https://data.mendeley.com/datasets/c3pvc272df/2

## Value of the Data

• Basic sexual dimorphism and reproductive biology information on this high value tropical fish is still scarce. The data on morphometric measurements and feeding regime of *Tor tambroides* from juvenile to adults will helps on the husbandry, broodstock management and development of breeding technique for conservation, commercial, experimental and personal usage purpose.
• These data will benefit breeders, conservationist, scientist and ornamental hobbyist of this emerging aquaculture species.
• These data can be used by scientist and conservationist for studies on sex determination/dimorphism of *T. tambroides* broodstocks, identifying the differences between wild and hatchery reared broodstocks, and comparing the population stock of *T. tambroides*, where all studies are focusing on the morphometric measurements.

## 1 Data Description

Raw data and mean of the morphometric measurements on the weight and morphometric characteristics of *T. tambroides* are provided in the Mendeley dataset [1]. The Shapiro-Wilk normality test showed that all the data are normally distributed (*p* > 0.05). Hotelling's T^2^ analysis conducted showed that there was a statistically significant difference between the morphometric measurements of male and female *T. tambroides* broodstocks reared in captivity F(28, 25) = 5.737, *P* < 0.0005; Wilks' Λ = 0.135; partial η^2^ = 0.865. Six morphometric measurements were found to have a significant difference between male and female captive reared broodstock (*p* < 0.0005), namely measurement 1, 3, 10, 16 and 21 for Truss network measurements and head length for conventional measurement. The mean values of all six measurements are shown in Table 1.

**Table 1:** Mean measurements of conventional and Truss network morphometric parameters where significant difference were found between male and female *T. tambroides* broodstock in captivity.

Morphometric characteristics	Measurements	Female (cm)	Male (cm)
Conventional Morphometric	Head Length (HL)	9.47 ± 0.574	8.99 ± 0.477
Truss Network	1	8.55 ± 0.598	8.03 ± 0.357
	3	7.82 ± 0.447	7.44 ± 0.282
	10	3.21 ± 0.465	2.73 ± 0.287
	16	5.03 ± 0.601	6.07 ± 0.401
	21	7.40 ± 0.568	8.14 ± 0.481

## 2 Experimental Design, Materials and Methods

### 2.1 Location of study, sample source and rearing setup

The study was conducted at the Mahseer Hatchery, Faculty of Fisheries and Food Sciences, Universiti Malaysia Terengganu. Twenty-seven males (TL: 53.88 ± 2.86 cm; W: 1.82 ± 0.23 kg) and 27 females (TL: 53.97 ± 3.62 cm; W: 1.86 ± 0.35 kg) broodstock were examined in this study. The fish cohort were bought from a local commercial supplier while they were five-inch fingerlings and were raised and reared for five years at the hatchery. The sex of each broodstocks were confirmed through abdomen hand stripping and the presence of tubercles on the operculum area. Mature males possessed tubercles and were able to shed sperm when its abdomen is gently pressured while tubercles were absent in female broodstocks and they excrete eggs or did not excrete anything [2].

A recirculating aquaculture system was used to rear all the broodstocks where they were housed in five-ton tanks at the stocking density of four fish/ton (M:F = 1:1). Water parameter (salinity, temperature and ammonia) was monitored daily and maintained at 0ppt, 26–28 ˚C and 0ppm NH3. The fish were fed twice daily till satiation. The feeding regime throughout the five years rearing are shown in Table 2.

**Table 2:** Feed used throughout the five years captive rearing.

Life stage	Type of commercial feed
Starter (fish size: <300 g) The first 8 months in captivity	• Floating (3.3 mm) (32% crude protein) • Starter (2 mm) (32% crude protein and 4% crude fat)
Grower (fish size: 300 g to 1 kg) Eight months to 2 years in captivity	• Floating pellet no 4 and 5 (40% crude protein and 5% crude fat)
*Finisher (fish size: >1 kg) Two till five years in captivity	• Floating pellet no 6 (40% crude protein and 5% crude fat) • Floating Pellet no 6(10–11 mm) (43% min crude protein and 8% crude fat) • Spirulina Enriched Floating Fish Food XL(6–7 mm) (48% min crude protein and 3% crude fat)

*Feed were changed from those with the lowest protein to the highest protein content to promote growth and maturation.

### 2.2 Morphometric measurements

Since the broodstocks have high value, limited and are being used in breeding programs, morphometric measurements were conducted using digital image analysis. All the fish were anesthetized using clove oil (30ppm) before their image was captured using a 14.2-megapixel digital single lens reflex (DSLR) camera (NIKON D3100, Japan) fitted with a midrange zoom lens (NIKON AF-S DX Nikkor 18–55 mm f/3.5–5.6G VR II, Japan). For standardization, each of the broodstocks images were taken on the left side of their body where a ruler was positioned underneath and the distance between the camera and samples were set at 1.2 m. The free ImageJ [3] software was then used to measure the morphometric characteristics.

**Fig.1:**
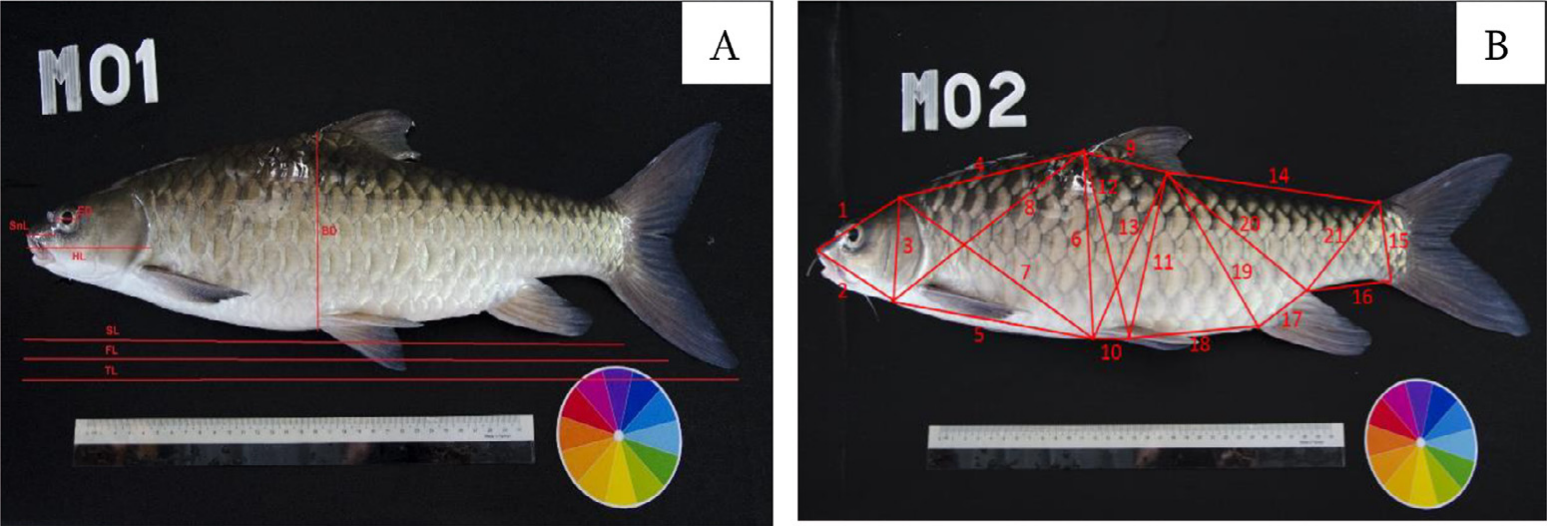
Morphometric characteristics used in this study [3]. A) Conventional morphometric characteristics B) Truss network system.

Altogether there were 28 morphometric characteristics measured in this study where they were chosen based on those used previously for *Tor tambroides* [4]. The morphometric characters include seven conventional and 21 Truss network system characteristics as shown in Fig. 1.

### 2.3 Data analysis

Hotelling's *T*^2^ statistical analysis were used in this study to determine the differences between the morphometric characteristics of each sex. This analysis was chosen since there were only two group of the independent variables (male and female) hence it is considered a special case of the one-way multivariate analysis of variance (one-way MANOVA) [5]. Furthermore, this analysis is also an extension of the independent-samples t-test to incorporate two or more dependent variables, in this case the 28 morphometric characteristics. Preliminary analysis which includes Shapiro-Wilk normality test, boxplot and Mahalanobis distance (to detect univariate and multivariate outliers), scatterplot (to identify linear relationship between all data) and a Box's M test (to detect homogeneity of variance-covariance matrices) were conducted prior to running the Hotelling's *T*^2^ analysis. A Bonferroni adjusted α level of 0.001785 with a simultaneous 95% confidence level was set [5]. Differences were considered to be significant at *P* < 0.0005. All analyzed data are presented as mean ± standard deviation unless otherwise stated. All data were analyzed using IBM SPSS software version 25.

## Ethics Statement

The authors confirm that all experiments comply with the ARRIVE guidelines and were carried out in accordance with the U.K. Animals (Scientific Procedures) Act, 1986 and associated guidelines, EU Directive 2010/63/EU for animal experiments, or the National Institutes of sentence spacing?

Health guide for the care and use of Laboratory animals (NIH Publications No. 8023, revised

1978)].

Moral and ethical aspect of the research such as animal handling and minimum amount of fish needed for valid statistical analysis also complied with the Research Ethics Guidelines of sentence spacing?

Universiti Malaysia Terengganu.

## CRediT Author Statement

**Abduh:** Conceptualization, Methodology, Investigation, Validation, Writing – Original Draft **Norazmi-Lokman**: Conceptualization, Methodology, Formal analysis, Writing – Original Draft **Gusti Afiz, Nurul Hayati, Syahnon**: Methodology, Investigation **Abol-Munafi**: Funding Acquisition, Supervision, Resources, Writing – Review&Editing

## Declaration of Competing Interest

The authors declare that they have no known competing financial interests or personal relationships which have, or could be perceived to have, influenced the work reported in this article.

## References

[1] Norazmi-Lokman N.H., Abduh M.Y. (2020). Raw data on the morphometric measurements of Malaysian Mahseer (*Tor tambroides*) in captivity. Mendeley Data.

[2] Desai V.R. (2003). Synopsis of Biological Data on the Tor Mahseer *Tor tor* (Hamilton, 1822) (No. 158).

[3] Collins T.J. (2007). ImageJ for microscopy. Biotechniques.

[4] Pollar M., Jaroensutasinee M., Jaroensutasinee K. (2007). Morphometric analysis of *Tor tambroides* by stepwise discriminant and neural network analysis. Int. J. Bioeng. Life Sci..

[5] Laerd Statistics, Hotelling's T2 using SPSS Statistics. Statistical tutorials and software guides. https://statistics.laerd.com/, 2017 (Accessed 5 March 2020).

